# Immersive virtual reality-based rehabilitation for subacute stroke: a randomized controlled trial

**DOI:** 10.1007/s00415-023-12060-y

**Published:** 2023-11-10

**Authors:** Qianqian Huang, Xixi Jiang, Yun Jin, Bo Wu, Andrew D. Vigotsky, Linyu Fan, Pengpeng Gu, Wenzhan Tu, Lejian Huang, Songhe Jiang

**Affiliations:** 1 Department of Rehabilitation Medicine, Rehabilitation Medicine Center, The Second Affiliated Hospital and Yuying Children’s Hospital of Wenzhou Medical University, Wenzhou, 325027 Zhejiang China; 2 Integrative and Optimized Medicine Research Center, China-USA Institute for Acupuncture and Rehabilitation, Wenzhou Medical University, Wenzhou, 325027 Zhejiang China; 3 Department of Information, The Second Affiliated Hospital and Yuying Children’s Hospital of Wenzhou Medical University, Wenzhou, 325027 Zhejiang China; 4 Departments of Biomedical Engineering and Statistics, Northwestern University, Evanston, IL 60208 USA; 5 Department of Radiology, The Second Affiliated Hospital and Yuying Children’s Hospital of Wenzhou Medical University, Wenzhou, 325027 Zhejiang China; 6 Department of Neuroscience, Feinberg School of Medicine, Northwestern University, Chicago, IL 60611 USA

**Keywords:** Immersive virtual reality rehabilitation, Stroke, Functional magnetic resonance imaging, Brain functional connectivity

## Abstract

**Objective:**

Few effective treatments improve upper extremity (UE) function after stroke. Immersive virtual reality (imVR) is a novel and promising strategy for stroke UE recovery. We assessed the extent to which imVR-based UE rehabilitation can augment conventional treatment and explored changes in brain functional connectivity (FC) that were related to the rehabilitation.

**Methods:**

An assessor-blinded, parallel-group randomized controlled trial was performed with 40 subjects randomly assigned to either imVR or Control group (1:1 allocation), each receiving rehabilitation 5 times per week for 3 weeks. Subjects in the imVR received both imVR and conventional rehabilitation, while those in the Control received conventional rehabilitation only. Our primary and secondary outcomes were the Fugl-Meyer assessment’s upper extremity subscale (FMA-UE) and the Barthel Index (BI), respectively. Both intention-to-treat (ITT) and per-protocol (PP) analyses were performed to assess the effectiveness of the trial. For both the FMA-UE/BI, a one-way analysis of covariance (ANCOVA) model was used, with the FMA-UE/BI at post-intervention or at follow-up, respectively, as the dependent variable, the two groups as the independent variable, baseline FMA-UE/BI, age, sex, site, time since onset, hypertension and diabetes as covariates.

**Results:**

Both ITT and PP analyses demonstrated the effectiveness of imVR-based rehabilitation. The FMA-UE score was greater in the imVR compared with the Control at the post-intervention (mean difference: 9.1 (95% CI 1.6, 16.6); *P* = 0.019) and follow-up (mean difference:11.5 (95% CI 1.9, 21.0); *P* = 0.020). The results were consistent for BI scores. Moreover, brain FC analysis found that the motor function improvements were associated with a change in degree in ipsilesional premotor cortex and ipsilesional dorsolateral prefrontal cortex immediately following the intervention and in ipsilesional visual region and ipsilesional middle frontal gyrus after the 12-week follow-up.

**Conclusions:**

ImVR-based rehabilitation is an effective tool that can improve the recovery of UE functional capabilities of subacute stroke patients when added to standard care. These improvements were associated with distinctive brain changes at two post-stroke timepoints. The study results will benefit future patients with stroke and provide evidence for a promising new method of stroke rehabilitation.

**Trial registration:**

ClinicalTrials.gov identifier: NCT03086889.

**Supplementary Information:**

The online version contains supplementary material available at 10.1007/s00415-023-12060-y.

## Introduction

Upper extremity (UE) motor impairment, including loss of movement, sensation, and dexterity, is a common manifestation of patients after stroke [1, 2], compromising patients’ independence in daily activities, thus remarkably diminishing their quality of life. There have been few effective treatments to improve UE function after stroke. Moreover, conventional rehabilitation therapy techniques, including motor relearning, proprioceptive neuromuscular facilitation, and neurodevelopmental therapy [3, 4] are tedious and resource-intensive [5]. Thus, developing novel interventions to improve UE function after stroke is clinically important [6].

Immersive VR-based program (imVR) training is a promising treatment in that it can enhance motor recovery by providing high-intensity, highly repetitive, and task-orientated training [7], usually unachievable by conventional rehabilitation therapies due to its unique features: (1) personalized treatment with a variety of training options that are interesting and enjoyable[8]; (2) alternative relaxing environments that emulate reality, allowing patients to relearn motor functions in a safe environment, and (3) intuitive and easy to operate, in turn boosting the transferability of the skills learned in the virtual environment into real life [9].

So far, the use of imVR systems for UE motor rehabilitation has not yet been sufficiently studied or implemented. While a few studies have applied imVR in stroke rehabilitation training and demonstrated that it could improve the effectiveness of UE rehabilitation training in stroke patients [10, 11], most of these studies have small sample sizes (≤ 10 patients, and even a single case) and lack control groups, only focus on short-term effects, and seldom investigate the underlying changes in brain activity [12–14]. Additionally, studies suggest that recovery processes plateau after about 6 months [15]; neuroplasticity may have become less elastic in this timeframe, so patients in the subacute phase (7 days–6 months post-stroke) may benefit from imVR therapies more than in chronic phases of stroke [16]. Thus, randomized experiments with parallel control groups are necessary to compare the effectiveness of imVR with conventional therapy on UE motor recovery in stroke patients in their subacute phase.

## Materials and methods

### Study design

In this study, we hypothesized that imVR rehabilitation for patients with subacute stroke would augment UE motor recovery compared to conventional therapy and that this improvement would correlate with brain neurophysiological change. To assess these hypotheses, we enrolled 40 patients in a single-blind, parallel-group, and randomized trial, during which resting-state functional MRI (RS-fMRI) was used to investigate neuroplasticity resulting from rehabilitation [17] and the relationship between changes in brain functional connectivity and recovery of UE motor performance was explored afterward.

### Ethical procedure

This study was performed at the Second Affiliated Hospital and Yuying Children’s Hospital of Wenzhou Medical University, China from March 2017 to July 2021. The study was approved by the Institutional Review Board of the Hospital (No. 2017LCKY-09) and all participants provided written informed consent. The clinical trial was registered at ClinicalTrials.gov (NCT03086889) and its original study protocol has been published in [18].

### Subject enrollment and allocation

**Fig. 1:**
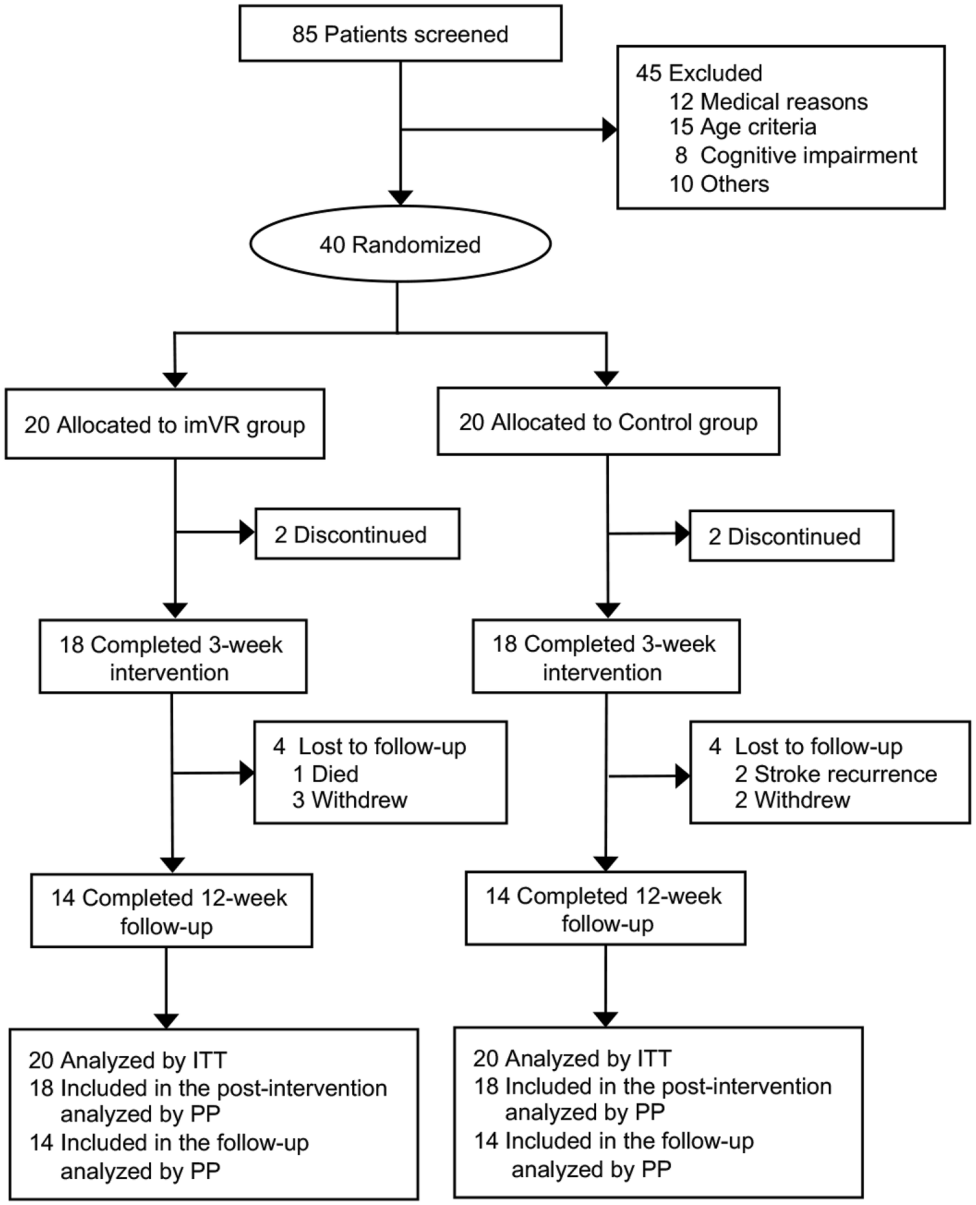
CONSORT diagram of study enrollment. *imVR* immersive virtual reality, *ITT* intention to treat, *PP* per-protocol

As shown in Fig. 1, 85 stroke patients with subcortical lesions in their subacute stage were screened, and 40 subjects were enrolled. The enrolled subjects were randomly evenly allocated into a new rehabilitation treatment with an imVR system (imVR group) or a conventional treatment program (Control group). Each subject was randomly assigned a code based on computer-generated, permuted block randomization with a block size of 4. Because of the nature of the intervention, subjects and therapists could not be blinded to the allocated treatment. These therapists did not participate in assessments of the outcomes. Demographic and baseline clinical characteristics and the largest area of lesion of both groups are summarized in Table 1 and marked by a yellow arrow in Supplementary Fig. 1.

**Table 1:** Demographic and baseline clinical characteristics of enrolled 40 subjects

Characteristic	imVR (*n* = 20)		Control (*n* = 20)		*P* value^a^
Age, mean (SD), y	63.3	(14.3)	65.1	(6.1)	0.620
Sex, male, NO. (%)	13	(65)	11	(55)	0.519
Right handedness, NO. (%)	20	(100)	20	(100)	1.000
Time since stroke, mean (SD), d	18.8	(8.4)	19.0	(6.6)	0.934
Hypertension, NO. (%)	13	(65)	16	(80)	0.288
Diabetes, NO. (%)	11	(55)	12	(60)	0.749
Stroke type, NO. (%)					
Ischemia	19	(95)	18	(90)	1.000
Hemorrhage	1	(5)	2	(10)	
Side of brain lesion, NO. (%)					
Right	10	(50)	13	(65)	0.337
Left	10	(50)	7	(35)	
Stroke location, subcortical, NO. (%)	20	(100)	20	(100)	1.000
FMA-UE, mean (SD)	17.5	(12.8)	14.8	(13.0)	0.172
BI, mean (SD)	56.0	(17.4)	47.5	(21.0)	0.504

*SD* standard deviation, *imVR* immersive Virtual Reality, *FMA-UE* Fugl–Meyer Assessment-Upper Extremity, *BI* Barthel Index

^a^*P* values show the consistency of the baseline data with the null hypothesis due to randomization; they are not intended to be interpreted inferentially [19]

### Inclusion and exclusion criteria

The inclusion criteria were as follows: to be eligible, subjects must (1) be over 30 but less than 85 years old; (2) have had their first stroke within the past month; (3) be in the subacute stage with a subcortical lesion location including the basal ganglia, internal capsule, corona radiata or brainstem; and (4) have a starting upper-limb function of Brunnstrom stage II–IV. The exclusion criteria were as follows: (1) history of transient ischemic attack (TIA); (2) failure of critical organs, such as heart, lung, liver, and kidney; (3) previous history of brain neurosurgery or epilepsy; (4) severe cognitive impairments or aphasia (incapable of understanding the instructions given by therapists); (5) not suitable for an MRI scan; and (6) enrollment in another clinical trial involving physical therapy or an investigational drug.

### Intervention design

Subjects received assessments at three-time points: immediately after randomization (baseline, week 0), immediately following the conclusion of the randomized rehabilitation program (post-intervention, week 3), and follow-up 12 weeks after concluding the rehabilitation program (follow-up, week 15). The assessments include MRI scans and evaluations performed by assessors who were blinded to group allocation in the whole study and with at least 2-years of experience in physical therapy.

Subjects in the Control received a 60-min conventional rehabilitation program per day. Conventional rehabilitation was designed with similar intensity and complexity to simulate the skills required in the immersive VR group. This conventional rehabilitation program consists of physical and occupational therapy, including grips and selective finger movements, gross movement, strength training, stretching, and training in activities of daily life. In contrast, subjects in the imVR received the first 30 min of conventional rehabilitation, and in the second 30 min, the rehabilitation was performed in imVR systems. The details of the imVR systems were introduced in [18]. Subjects in the imVR group were required to complete 6 programs (Supplementary Fig. 4): frying dumplings and noodles by controlling a wok handle in a virtual kitchen; popping balloons by controlling a sword in a virtual fencing hall; punching dolls by controlling a big fist in a virtual boxing arena; playing basketball in a virtual court, in which the ball is shot by a controller and the height and distance is varied over time; collecting eggs into a virtual basket by a controller; and tidying up a desk and moving objects to a designated position in a virtual office. All subjects received rehabilitation training 5 days per week over 3 weeks.

In the early stages of rehabilitation, due to the poor function of the hemiplegic side upper limb, subjects had to complete the imVR programs with the help of the limb on the unaffected side. With the recovery of the hemiplegic side upper limb function, the subjects independently completed the 6 games with only their limb on the hemiplegic side.

### Outcomes

The upper extremity portion of the Fugl-Meyer assessment (FMA-UE) [20] and the Barthel Index (BI) [21] were the primary and secondary outcome measures for this trial, respectively. The FMA-UE, which measures arm movement ability across several domains (motor function, balance, sensation, range of motion, and pain), is a standard clinical tool for evaluating changes in motor impairment after stroke. The BI measures activities of daily living (ADL), consisting of feeding, grooming, bathing, bowel control, chair transfer, bladder control, toileting, dressing, ambulation, and stair climbing. RS-fMRI was an additional outcome measure. Degree, a derivative parameter derived from brain functional connectivity (FC), was used to assess neurobiological correlates of imVR-based rehabilitation and to relate changes in brain activity to motor recovery.

### MRI data acquisition

All subjects were scanned on a 3.0 T GE-Discovery 750 scanner with the following parameters: for anatomical T1-MRI data: TR/TE = 7.7/3.4 ms, flip angle = 12°, FOV = 256 × 256 mm^2^, resolution = 256 × 256, number of slices = 176, isometric voxel size = 1 × 1 × 1 mm^3^; for fMRI data: TE/TR = 30/2500 ms with interleaved ordering, voxel size = 3.4375 × 3.4375 × 3.5 mm^3^, in-plane resolution = 64 × 64, number of volumes = 230, and flip angle = 90°.

### RS-fMRI data quality control, preprocessing, and registration

Mean framewise displacement (mFD) of each RS-fMRI data set, calculated as the sum of mean displacement along 6 dimensions, indicating the extent of head motion over the duration of the scan, was used as a metric for quality control of RS-fMRI data and a covariate in the further statistical analysis.

A similar preprocessing pipeline to [22] was applied to all RS-fMRI data. Briefly: removal of the first four volumes (10 s) for magnetic field stabilization; motion correction; slice-time correction; intensity normalization; high-pass temporal filtering (0.008 Hz) for correcting low-frequency signal drift; nuisance regression of 6 motion vectors, signal-averaged overall voxels of the eroded white matter and ventricle regions, and global signal of the whole brain; motion-volume censoring by detecting volumes with an FD larger than 0.5 mm, Derivative Variance Root mean Square after *Z* normalization larger than 2.3, and standard deviation after *Z* normalization larger than 2.3, and scrubbing above detected (volume = *i*) and adjacent four volumes (*i* − 2, *i* − 1, *i* + 1, *i* + 2) [23, 24]; band-pass filtering (0.008–0.1 Hz) by applying a 4th-order Butterworth filter.

All pre-processed RS-fMRI data were registered to the MNI152 template using a two-step procedure, in which the mean of preprocessed fMRI data was registered with a 7-degree-of-freedom affine transformation to its corresponding T1 brain (FLIRT); transformation parameters were computed by nonlinearly registering individual T1 brain to the MNI152 template (FNIRT). Combining the two transformations by multiplying the matrices yielded transformation parameters to normalize the pre-processed fMRI data to the standard space. All the final registered images were manually examined.

After the registration, for those subjects who had left-sided lesions, the registered images were flipped from left to right along the midsagittal line. In the end, the right side corresponded to the ipsilesional hemisphere.

### Resting-state functional connectivity network

For each subject, their RS brain functional connectivity networks (FCN) across gray matter were generated. First, the blood oxygenation level-dependent (BOLD) signal was extracted from each gray matter voxel in the preprocessed and registered RS-fMRI data. Following this, we calculated voxel-based pairwise Pearson correlation coefficients of BOLD signals to construct a correlation matrix, which was then Fisher’s *z* transformed. To normalize the variation of the strength of brain FCN across individuals, a link density—the percentage of links with respect to the maximum number of possible links—was predetermined, corresponding to a correlation threshold [25, 26]. In our study, 10% link density was applied. Consequently, an indirectly connected brain FCN was generated after the correlation matrix was binarized by the subject-dependent threshold to create an adjacency matrix.

### Degree comparison and associations between changes in degree and motor recovery

Derived from the brain FCN, one of brain network topological measurements, degree—a measure of network hubness [27], was used to investigate the effect of imVR rehabilitation training on brain FCN. For each voxel on the gray matter, its degree equals the number of links to the other gray matter voxels except those within two adjacent voxels to mitigate the effects of motion [28, 29]. This voxel-wise degree indicates the relative strength of local neural activity within a subject’s brain FCN.

To compare degree maps between the imVR and the Control groups, we used a general linear model (GLM) with the degree at post-intervention and at follow-up, respectively, as the dependent variable, the two groups as the independent variable, baseline degree, age, sex, side of brain lesion, time since stroke, hypertension, diabetes, and mFD as confounds; family-wise cluster correction (*t* > 3.5, *P* < 0.01) [30] was applied afterward.

For each statistically significant cluster between the imVR and the Control, we compared the average degree count extracted from the cluster and correlated changes in the average degree with the recovery of UE motor performance.

### Network reorganization

After identifying brain clusters that statistically significantly differed between the imVR and the Control groups, we explored to which regions these significant clusters connected and if these connected regions were also statistically significantly different (reorganized) between groups. The analysis was run in network (module) space, spanned by 333 cortical parcels defined in [31] and 16 in-house-defined subcortical regions (total 349 regions), from which 13 functional networks are constructed: visual, auditory, default-mode, cingulo-opercular task control, fronto-parietal task control, sensory/somatomotor mouth, sensory/somatomotor hand, dorsal attention, ventral attention, subcortex, salience, cinguloparietal, retrosplenial temporal [31]. For each subject, the status of functional connectivity between each significant cluster and the 349 parcels (regions) was set to as “*connected*” if their correlation coefficients were greater than the subject-dependent threshold (expounded in Resting-state functional connectivity network) or “*disconnected*” if less, generating a vector of 349 connection status. Chi-squared test was independently applied to each parcel to determine if there existed a statistically significant difference in connection status between the imVR and the Control groups (*P* < 0.05). The results were reported in circular plots.

### Statistical and data analyses

In our previous sample size calculation [18], we estimated that 30 subjects per group would be sufficient to assess the effectiveness of the imVR training given a two-tailed comparison and set the type I error rate at 0.05 with 80% power and effect size of 0.75. In our protocol, one interim analysis was planned after 60% of subjects completed the post-intervention. If the *P* value corresponding to the effectiveness of the imVR (FMA-UE) was less than 0.025, the trial would be terminated earlier. We had the interim analysis when the number of subjects per group reached 20 and found the *P* value is 0.019 (Table 2) so the recruitment stopped earlier.

**Table 2:** Outcomes at Baseline, Post-intervention, and Follow-up by Groups

	imVR (*n* = 20)	Control (*n* = 20)	Between group comparisons	
			Mean difference (95% CI)	*P* value
FMA-UE, mean (SD)				
Baseline	17.5 (12.8)	14.8 (13.0)	2.7 (− 5.5 to 11.0)	
Post-intervention	34.9 (18.4)	24.0 (20.7)	9.1^a^ (1.6 to 16.6)	0.019^b^
Follow-up	48.0 (15.1)	36.0 (20.0)	11.5^a^ (1.9 to 21.0)	0.020^b^
BI, mean (SD)				
Baseline	56.0 (17.4)	47.5 (21.0)	8.5 (− 3.9 to 20.9)	
Post-intervention	85.3 (12.2)	72.8 (21.7)	8.3^a^ (0.08 to 16.5)	0.048^b^
Follow-up	98.8 (3.4)	92.2 (8.2)	4.8^a^ (0.85 to 8.8)	0.019^b^

*imVR* immersive virtual reality, *CI* confidence interval, *FMA-UE* Fugl–Meyer Assessment-Upper Extremity, *SD* standard deviation, *BI* Barthel Index; ^a^Adjusted estimates after controlling for baseline FMA-UE score or BI score, age, sex, site, time since onset, hypertension and diabetes; ^b^*P* value was determined using ANCOVA model with baseline score, age, sex, side of brain lesion, time since stroke, hypertension and diabetes as covariates of no interest

Both intention-to-treat (ITT) and per-protocol (PP) analyses were performed to assess the effectiveness of the trial. The ITT analysis was conducted with all randomly assigned participants included in the analysis, applying the Markov Chain Monte Carlo method with linear regression (only FMA-UEs or BIs as predictors in the model) for the imputation of any missing value (20 subjects in imVR vs. 20 subjects in Control). The PP analysis included participants who had at least a 2-week long intervention (18 vs. 18 post-intervention and 14 vs. 14 follow-up).

This randomized controlled trial is a two-group independent design examining the effects of imVR on the rehabilitation of patients with subacute stroke and the assessments were repeated three times. We were interested in the change of outcomes (i.e., recovery) between the two groups. So, for both the FMA-UE/BI, a one-way analysis of covariance (ANCOVA) model was used, with the FMA-UE/BI at post-intervention or at follow-up, respectively, as the dependent variable, the two groups as the independent variable, baseline FMA-UE/BI, age, sex, site, time since onset, hypertension and diabetes as covariates. *P* < 0.05 was statistically significant.

To investigate the effects of imVR on brain activity both at post-intervention and the follow-up, for each time point the GLM model was applied with a degree as the dependent variable, two groups as the independent variable, base-line degree, age, sex, site, time since onset, hypertension, diabetes, and *log*(mFD) as covariates; cluster-correction was performed afterward (*t* > 3.5, *P* < 0.01) [30].

Pearson correlations were performed to examine how mean degree, extracted from the significant cluster, tracks with changes of outcomes (FMA-UE or BI) using (1) post-intervention and baseline and (2) follow-up and baseline, respectively. *P* < 0.05 was statistically significant.

Independent *t* tests and Mann–Whitney *U* tests were employed to compare outcomes that did and did not meet residual normality assumptions, respectively, between the two groups. The Chi-square test was used to compare categorical outcomes.

A mixed effects model was used to assess the significance of difference in brain motion during scanning, represented by *log*(mFD), across three assessments between the imVR and the Control groups. *P* < 0.05 was statistically significant.

## Results

### Upper extremity motor performance

The ITT analysis demonstrated that the primary outcome, FMA-UE score, was statistically significantly greater in the imVR group compared with the Control group both at the post-intervention (adjusted effect: 9.1, 95% CI (1.6–16.6); *P* = 0.019) and at the follow-up assessment (adjusted effect: 11.5, 95% CI (1.9–21.0); *P* = 0.020) (Table 2); the secondary outcome, BI score, was also statistically significantly greater in the imVR (Table 2). The PP analysis presented the significance of the imVR group as well and was detailed in Supplementary Table 1.

### Post-intervention RS-fMRI results

**Fig. 2:**
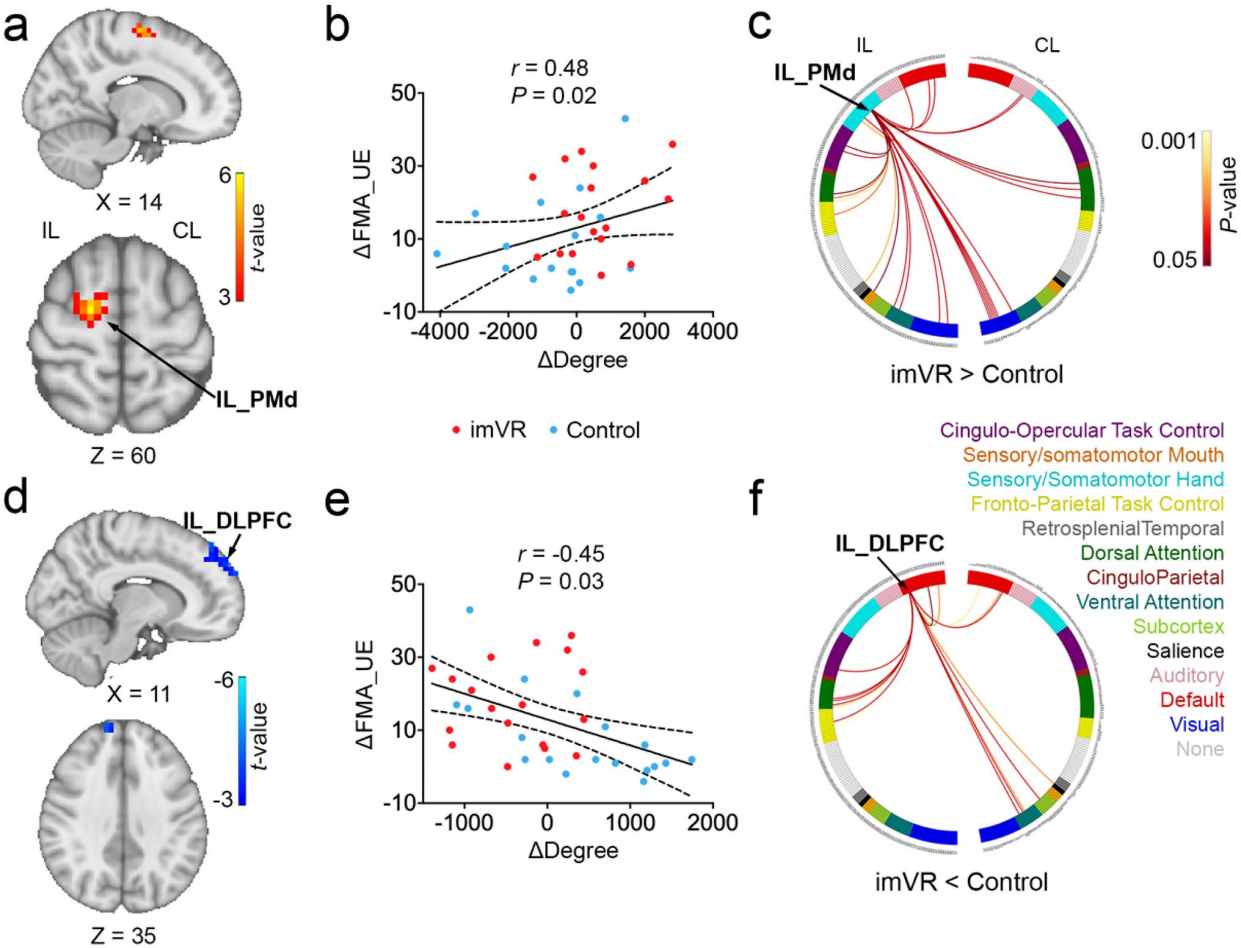
Changes in degree in IL_PMd and DLPFC were Significantly Associated with Recovery of Motor Performance at the Post-intervention. **a** The imVR had a greater degree of IL_PMd at the end of the intervention (week 3) compared with the Control. **b** A positive correlation between the change in degree in IL_PMd and the change of FMA-UE from baseline to post-intervention. **c** The circular plot shows the difference in functional connections to IL_PMd between the imVR and Control groups (*P* < 0.05) in the network space. The IL_PMd region is assigned to the sensory/somatomotor hand network. **d** The imVR presented a lower degree in IL_ DLPFC at the end of the intervention. **e** Change in mean degree in IL_DLPFC correlated with change of FMA-UE from baseline to post-intervention. **f** The circular plot shows the difference in functional connections to IL_DLPFC, which is assigned to the default-mode network. Cluster correction was performed with* t* > 3.5, *P* < 0.01. ΔDegree and ΔFMA-UE are defined as the difference of degree and of FMA-UE, respectively, between post-intervention and baseline; *IL* ipsilesional, *CL* contralesional, *PMd* Dorsal Premotor Cortex, *DLPFC* Dorsolateral Prefrontal Cortex

Compared to the Control, the imVR exhibited greater degrees in Ipsilesional Dorsal Premotor Cortex (IL_PMd) (*P* = 0.008) and Ipsilesional Primary Motor Cortex (IL_M1) (*P* = 0.003) (Fig. 2a; Supplementary Table 2 and Supplementary Fig. 2a), and lower degrees in Ipsilesional and Contralesional Dorsolateral Prefrontal Cortex (IL_and CL_DLPFC) (*P* = 0.003; *P* < 0.001) at the end of intervention (Fig. 2d; Supplementary Table 2 and Supplementary Fig. 2b).

As shown in Fig. 2b and e, it was also revealed that the change of mean degree in IL_PMd and IL_DLPFC between the post-intervention and the baseline is, respectively, positively and negatively correlated with changes in FMA-UE (*r* = 0.48, *P* = 0.02; *r* = − 0.45, *P* = 0.03), indicating that the change of degree in both IL_PMd and in IL_DLPFC is associated with recovery of motor performance after the intervention. Furthermore, in network space, as shown in Fig. 2c, for IL_PMd, which is assigned to the sensory/somatomotor hand network, most of the degree differences (more connections to IL_PMd in the imVR group) are from the sensory/somatomotor hand, visual, frontal-parietal task control, ventral attention, dorsal attention, default mode network (DMN), and cingulo-opercular task control networks on the ipsilesional hemisphere. For IL_DLPFC, as shown in Fig. 2f, which is assigned to DMN, most of the degree difference (more connections to IL_DLPFC in the Control group) are from the ventral attention and DMN on a contralesional hemisphere, and DMN, cingulo-opercular task control, frontal-parietal task control and dorsal attention network on the ipsilesional hemisphere.

### Follow-up RS-fMRI results

**Fig. 3:**
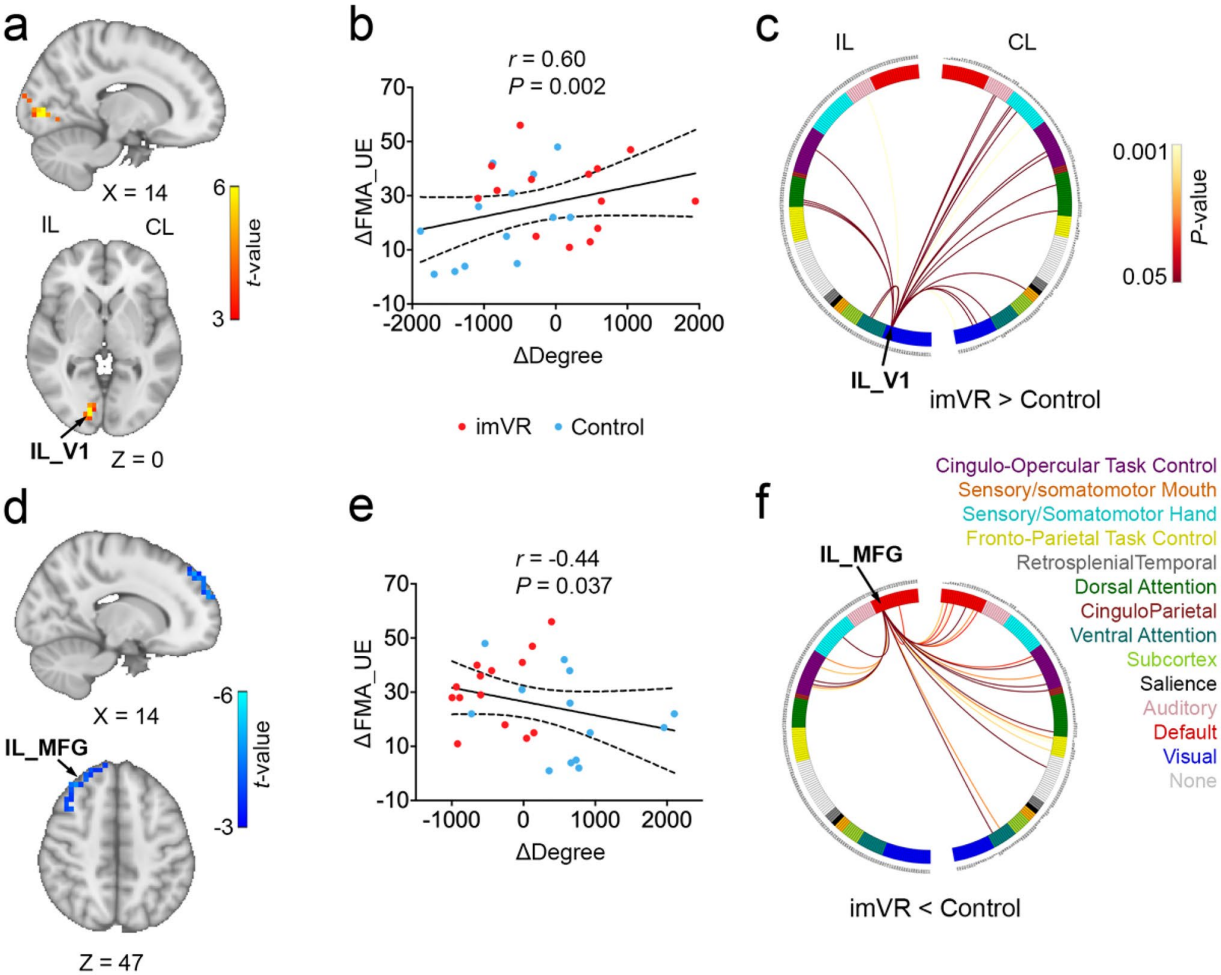
Changes in degree in IL_V1 and _MFG were Significantly Associated with Recovery of Motor Performance at the Fellow-up. **a** The imVR group had a greater IL_V1 degree at the end of the follow-up (week 15). **b** Changes in the mean degree of IL_V1 correlated with changes in FMA-UE from baseline to the end of the follow-up. **c** The circular plot shows the difference in functional connections to IL_V1 between the imVR and Control groups (*P* < 0.05) in the network space. The IL_V1 region is assigned to the Visual network. **d** imVR had a lower degree in the IL_MFG region at the end of the follow-up. **e** Mean IL_MFG degree negatively correlated between a change of mean degree in IL_MFG and the change of FMA-UE at the end of the follow-up. **f** The circular plot showed the difference in functional connections to IL_MFG between the imVR and Control groups (*P* < 0.05) in the network space. The IL_MFG region is assigned to the DMN network. cluster-correction was performed with* t* > 3.5, *P* < 0.01. ΔDegree and ΔFMA-UE are defined as the difference of degree and FMA-UE, respectively, between follow-up and baseline; *IL* Ipsilesional, *CL* contralesional, *V1* primary visual cortex, *MFG* middle frontal gyrus

At the end of the follow-up, the imVR exhibited greater degrees in Ipsilesional and Contralesional Primary Visual Cortex (IL_V1, CL_V1) (*P* = 0.002, *P* < 0.001), Contralesional Superior Parietal Gyrus (CL_SPG) (*P* < 0.001) and Ipsilesional Lateral Occipital Cortex (IL_LOC) (*P* < 0.001) (Fig. 3a; Supplementary Table 3 and Supplementary Fig. 3a), and the lower degree in Ipsilesional Middle Frontal Gyrus (IL_MFG) (*P* < 0.001), Ipsilesional Ventral Premotor Cortex (IL_PMv) (*P* = 0.004), Ipsilesional Inferior Frontal Gyrus (IL_IFG) (*P* < 0.001), Contralesional medial Prefrontal Cortex (CL_mPFC) (*P* < 0.001) and Contralesional Frontal Pole (CL_FP) (*P* < 0.001) regions as compared to the Control group (Fig. 3d; Supplementary Table 3 and Supplementary Fig. 3b). These 9 regions do not overlap with any of the 4 regions found to be statistically significant at the post-intervention timepoint.

It was also revealed changes in the mean degree of IL_V1 and IL_MFG from baseline to the end of the follow-up were positively and negatively correlated to change of FMA-UE (*r* = 0.60, *P* = 0.002; *r* = -0.44, *P* = 0.037), respectively, indicating that the changes in degree of IL_V1 and IL_MFG were associated with recovery of motor performance after the follow-up (Fig. 3b, e). Furthermore, in network space, for IL_V1, which is assigned to the visual network, most of the degree differences (more connections to IL_V1 in the imVR group) were from the somatosensory/somatomotor hand, visual, auditory, cingulo-opercular task control, dorsal attention and ventral attention networks on the contralesional hemisphere (Fig. 3c). For IL_MFG, which is assigned to DMN, most of the degree differences (more connections to IL_MFG in the Control group) were from the ventral attention, DMN, frontal-parietal task control, cingular-opercular task control networks on the contralesional hemisphere (Fig. 3f).

## Discussion

In this study, we demonstrated the effectiveness of imVR-based UE rehabilitation in stroke patients in their subacute phase. Participants assigned to the imVR training showed statistically significant improvements in UE motor impairment and daily living activity up to at least 12 weeks post-intervention and the magnitude of the improvements was much greater than other intervention programs [32–35]. Moreover, these improvements were associated with changes in degree derived from brain functional connectivity in ipsilesional premotor cortex and ipsilesional dorsolateral prefrontal cortex immediately after the intervention, and in ipsilesional visual region and ipsilesional middle frontal gyrus at the 12-week follow-up.

The clinical outcomes indicate that the imVR training has positive impacts on the recovery of UE function and activities of daily living (ADL) as assessed by the FMA-UE and BI, respectively. These beneficial effects may first be attributed to the imVR program with goal-orientated repetitive functional task practice, which encourages highly repetitive functional movements of the UE [36]. Repetition of task-specific movements is one of the fundamental principles of both motor learning and the production of cortical reorganization to improve motor function after a stroke [37, 38]. Animal studies demonstrate that a high number of repetitions are necessary to induce behavioral changes after brain injury, and, moreover, the amount of motor training correlates with motor recovery [39]. Similar evidence is also found in humans [40, 41]. ImVR provides immediate motor feedback and a strong sense of presence [42] during training, allowing for task-oriented repetitive exercises while changing the traditional treatment patterns which can feel boring for patients. These characteristics allow exercises to be repeated without causing fatigue and pain [41], leading to some potentially clinically important benefits compared with conventional rehabilitation and improvements in upper limb impairments that were translated into an improvement in ADL [43]. In addition, the imVR system itself may be beneficial for improving UE impairment, which provides an enriched environment (EE), thus exposing subjects to enhanced motor, sensory, cognitive, and social stimuli relative to a standard condition, which is also demonstrated by a clinical trial [44] and animal studies [45, 46].

Improvements in UE performance from the imVR training evaluated after the intervention correlated with reorganization of the brain networks; i.e., more brain functional connectivity to the sensory/somatomotor hand network, particularly on the ipsilesional side. After the 3-week intervention, 2 regions that statistically significantly differed between the imVR and the Control groups, IL-PMd (Fig. 2c) and -M1 (Supplementary Fig. 2d), ipsilesional and assigned to the sensory/somatomotor hand network, had more connections to them from ventral and dorsal attention, frontal-parietal and cingulo-opercular task control, and visual networks. This implies that the unique features of imVR—for example, more repetitive functional movements and attention—have enhanced motor planning and learning, visual stimuli, and motor control. This phenomenon is more greatly manifested in the region of IL_PMd, where connections from the sensory/somatomotor hand network itself are also increased and the change in degree was associated with UE motor recovery (Fig. 2b) and ADL (Supplementary Fig. 2c), consistent with previous studies [47, 48].

After the follow-up, the 3 significant regions in the visual network (IL_V1, CL_V1, and IL_LOC, Supplementary Fig. 3a) may play pivotal roles associated with improvements of UE motion recovery, in that the connections to the regions were increased and the change of degree between the follow-up and the baseline in the IL_V1 was associated with UE motor recovery. Studies suggested that V1 is involved in object recognition and representation, object localization, and vision-guided movement processing [49] and that LOC is functionally related to the hand area of primary somatosensory [50], which was shown in our previous study [51]. Compared with the Control, imVR may create more functional connections from the visual network to the sensory/somaomotor hand and cingulo-opercular task control network on the contralesional side at least 5 months after stroke, potentially enhancing a compensatory mechanism for loss of neuro activities on the ipsilesional side. Another region that has increased functional connectivity is SPG on the contralesional side (CL_SPG). Previous studies have shown that SPG, a hub for the exchange of sensory and motor-related information and critical for guiding upper limb movements toward the target and adjusting the shape of the hand to grasp the target [52], involved in the control of body movement, visual movement, oculomotor nerve activity and the guidance of visual spatial attention [53]. The imVR training may strengthen the function of SPG, promoting the improvement of patients' upper limb function after the imVR training guided patients to perform many repeated upper limb flexion and extension and grasping movements, while concurrently giving visual feedback.

The limitation of our study was that we could not blind subjects, limiting our ability to rule out possible placebo effects. A sham intervention may help to investigate whether the effects of the imVR rehabilitation are specific from the imVR training itself in that concealments of group allocation; however, such interventions are difficult to implement in stroke rehabilitation [54]. In addition, while distinct patterns of network reorganizations at two-time points were observed, we had not explored the mechanism for those significant brain regions, which absolutely will be our future work.

In conclusion, this study has demonstrated that imVR-based rehabilitation is a promising rehabilitation tool for improving the recovery of UE functional capabilities of subacute stroke patients and that these improvements are associated with distinct brain reorganization at two post-stroke stages.

### Supplementary Information

Below is the link to the electronic supplementary material.

Supplementary file1 (DOCX 9996 KB)

## Data Availability

All data will be shared upon reasonable request.
