# Supplementary material for: Immersive virtual reality-based rehabilitation for subacute stroke: a randomized controlled trial
Source: J Neurol. 2023 Nov 10;271(3):1256–66. doi: 10.1007/s00415-023-12060-y (PMC10896795; doi:10.1007/s00415-023-12060-y)
Supplement: Supplementary file 1 — Supplementary file1 (DOCX 9996 KB) [file 415_2023_12060_MOESM1_ESM.docx]

**Supplementary materials**

Immersive virtual reality-based rehabilitation for subacute stroke:

a randomized controlled trial

**Results 1.** Brain Image Motion

**Results 2.** Motor Recovery Performance with PP Analysis

**Results 3.** Supplementary Post-intervention fMRI Results of IL_M1 and CL_DLPFC

**Results 4.** Supplementary Follow-up fMRI Results of Other Regions

**Table 1.** Outcomes at Baseline, Post-intervention and Follow-up by Groups from per protocol analysis

**Table 2.** Brain Regions with Statistically Significant Degree Differences Between Baseline and Post-intervention at 10% Link Density

**Table 3.** Brain Regions with Statistically Significant Degree Differences Between Baseline and the End of Follow-up at 10% Link Density

**Figure 1.** The Largest Area of Lesion at the Axial Cross Section of Participants for Brain Image Analysis

**Figure 2**. Brain Properties at the End of the Intervention

**Figure 3**. Brain Properties at the End of Follow-up

**Figure 4.** Six imVR Programs

**Figure 5.** Comparison of Brain Motion Between the imVR and the Control Groups

**Results 1. Brain Image Motion**

For the RS-fMRI image quality, mFD of all subjects was less than 0.2 mm [1]; as shown in **Supplementary** **Fig. 5**, a mixed model determined that there was no statistically significant difference in brain motion during scanning, represented by *log*(mFD), across the three assessments between the imVR and the Control groups (*P* = 0.81).

**Results 2. Motor Recovery Performance with PP Analysis**

The PP analysis demonstrated that the primary outcome, FMA-UE score, was statistically significantly greater in the imVR group compared with the Control group both at the post-intervention (adjusted effect: 12.5, 95% CI (4.6 – 20.4); *P* = 0.003) and at the follow-up assessment (adjusted effect: 18.9, 95% CI (6.0 – 31.7); *P* = 0.006) (**Supplementary** **Table 1**).

Similarly, PP analyses showed that the secondary outcome, BI score, was also statistically significantly greater in the imVR group compared with the Control group both at the post-intervention (adjusted effects: 9.8, 95% CI (0.6 – 19.0); *P* = 0.038) and at the follow-up (adjusted effects: 6.7, 95% CI (0.5 – 12.8); *P* = 0.034).

**Results 3. Supplementary Post-intervention fMRI Results of IL_M1 and CL_DLPFC**

After cluster-correction (*t* > 3.5 and *P*< 0.01), the imVR group exhibited higher degree in IL_PMd (*P* = 0.008) and IL_M1 (*P* = 0.003) regions (**Fig. 2a**), and lower degree in IL_and CL_DLPFC (*P* = 0.003; *P* < 0.001) regions at the end of intervention compared to the Control group (Week 3) (**Supplementary** **Fig. 2b**). Post hoc analyses revealed that the change of mean degree in IL_PMd between post-intervention and baseline was positively correlated to the change of BI (*r* = 0.58, *P* = 0.004), indicating that the change of functional connectivity in IL_PMd was associated with recovery of activities of daily living (ADL) after the intervention (**Supplementary** **Fig. 2c**). Furthermore, in network space, for IL_M1 (**Supplementary** **Fig. 2d**), which is assigned to the sensory/somatomotor hand network, most of the degree difference (more connections to IL_M1 in the imVR group than in the Control group) are from sensory/somatomotor hand, ventral attention, dorsal attention, DMN, fronto-parietal task control and cingulo-opercular task control networks on the ipsilesional hemisphere. For CL_DLPFC (**Supplementary** **Fig. 2e**), which is assigned to the DMN, most of the differences (more connections to CL_DLPFC in the Control group than in the imVR group) were from ventral attention, fronto-parietal task control and DMN on the contralesional hemisphere, and DMN, cingulo-opercular task control and frontal-parietal task control network on ipsilesional hemisphere.

**Results 4. Supplementary Follow-up fMRI Results of Other Regions**

At the end of the follow-up (Week 15), after cluster-correction (*t* > 3.5 and *P*< 0.01), compared with the Control group, the imVR group exhibited higher degree in IL_V1 (*P* = 0.002), CL_V1 (*P* < 0.001), CL_SPG (*P* < 0.001) and IL_LOC (*P* < 0.001) ((**Supplementary** **Fig. 3a**), and lower degree in IL_MFG (*P* < 0.001), IL_PMv (*P* = 0.004), IL_IFG (*P* < 0.001), CL_mPFC (*P* < 0.001) and CL_FP (*P* < 0.001) regions at the end of follow-up (Week 15) (**Supplementary** **Fig. 3b**).

Furthermore, in network space, as shown in **Supplementary** **Fig. 3c**,for CL_V1 and IL_LOC regions, which are assigned to the visual network, and CL_SPG assigned to the dorsal attention network, most of the degree differences (more connections to CL_V1, CL_SPG and IL_LOC in the imVR than in the Control) are from the sensory/somatomotor hand, visual, auditory, cingulo-opercular task control, dorsal attention and ventral attention networks on the contralesional hemisphere. The IL_PMv and IL_IFG regions assigned to the dorsal attention network and most of the differences (more connections to IL_PMv and IL_IFG in the Control group than in the imVR group) are from the default, dorsal attention, cingular-opercular task and sensory/somatomotor hand networks on the contralesional hemisphere. CL_mPFC assigned to the default network and most of the differences (more connections to CL_mPFC in the Control group than in the imVR group) are from the ventral attention, DMN, fronto-parietal task control, cingular-opercular task control networks on the ipsilesional hemisphere. CL_FP assigned to the fronto-parietal task control network and most of the differences (more connections to CL_FP in the Control group than in the imVR group) are from the dorsal attention, default, fronto-parietal task control, cingular-opercular task control networks on the ipsilesional hemisphere.

**Table 1. Outcomes at Baseline, Post-intervention and Follow-up by Groups from per-protocol analysis**

Data are shown for the PP (per-protocol) analysis (18 vs. 18 post-intervention and 14 vs. 14 follow-up). FMA-UE, Fugl-Meyer Assessment-Upper Extremity; BI, Barthel Index; CI, Confidence Interval; imVR, immersive Virtual Reality; ^a^ *P* value was determined using independent samples *t*-test. ^b^ adjusted estimates after controlling for baseline FMA-UE score or BI score, age, gender, site, time since onset, hypertension and diabetes. ^c^ *P* value was determined using ANCOVA model with baseline score, age, sex, side of brain lesion, time since stroke, hypertension and diabetes as covariates of no interest.

**Table 2. Brain Regions with Statistically Significant Degree Difference Between Baseline and Post-intervention at 10% Link Density**

Abbreviations: PMd, Dorsal Premotor Cortex; M1, Primary Motor Cortex; DLPFC, Dorsolateral Prefrontal Cortex; IL, Ipsilesional; CL, Contralesional; MNI, Montreal Neurological Institute; imVR, immersive Virtual Reality. Note: ^a^Harvard-Oxford cortical and subcortical structural atlas; ^b^cluster-corrected (*t* > 3.5, *P*< 0.01).

**Table 3.** **Brain Regions with Statistically Significant Degree Differences Between Baseline and the end of Follow-up at 10% Link Density**

Abbreviations: V1, Primary Visual Cortex; SPG, Superior Parietal Gyrus; LOC, Lateral Occipital Cortex; MFG, Middle Frontal Gyrus; PMv, ventral premotor cortex; IFG, Inferior frontal gyrus; mPFC, medial Prefrontal Cortex; FP, Frontal Pole; CL, Contralesional; IL, Ipsilesional; MNI, Montreal Neurological Institute; imVR, immersive virtual reality. Note: ^a^Harvard-Oxford cortical and subcortical structural atlas; ^b^cluster-corrected (*t* > 3.5, *P*< 0.01).

**Figure 1** **The Largest Area of Lesion at the Axial Cross Section of Participants for Brain Image Analysis**


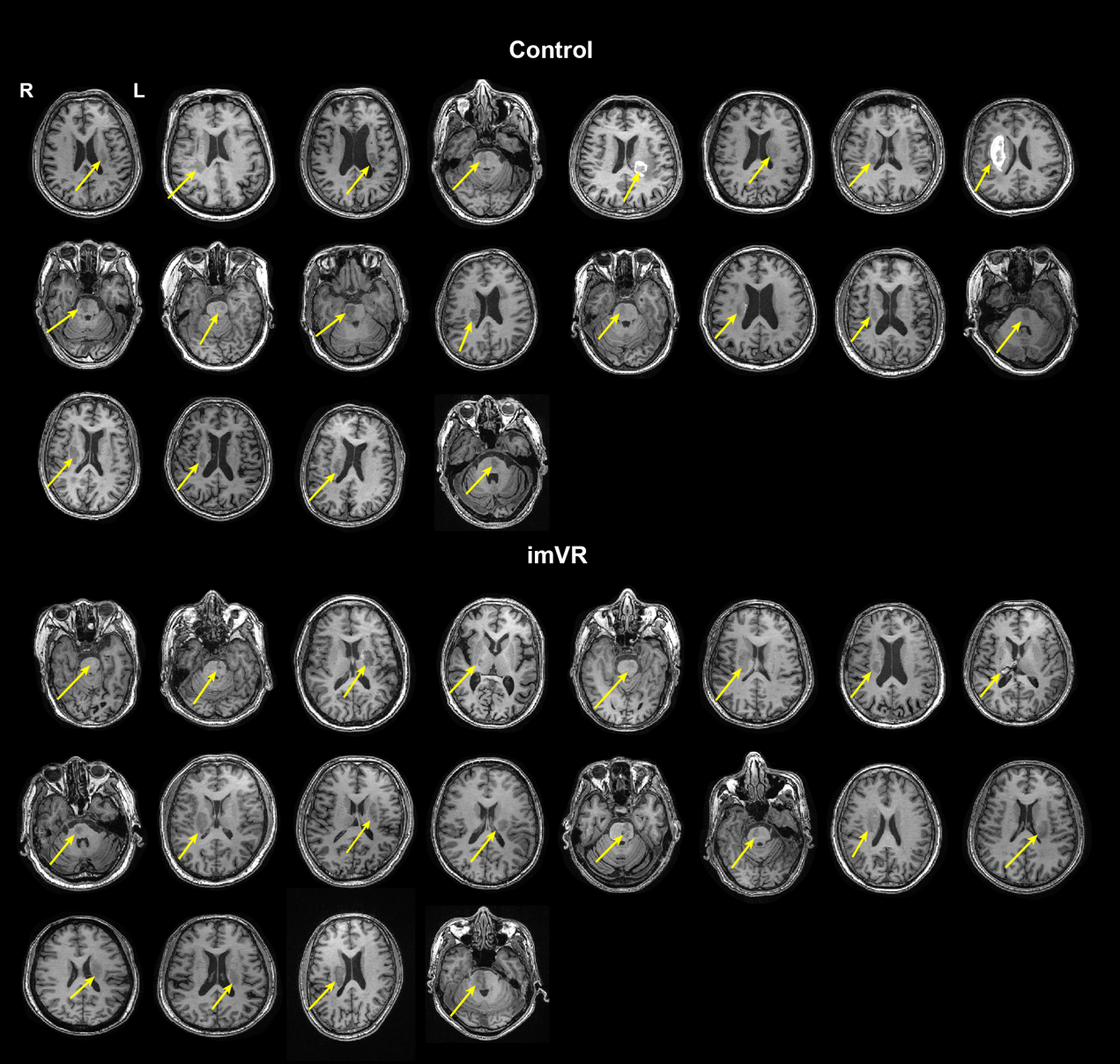


Yellow arrows indicate the largest lesion area of each stroke patient at the axial cross section for the imVR (bottom) and the Control (top) groups. R and L represent right and left hemisphere of brain, respectively.

**Figure 2 Brain Properties at the end of the Intervention**

**
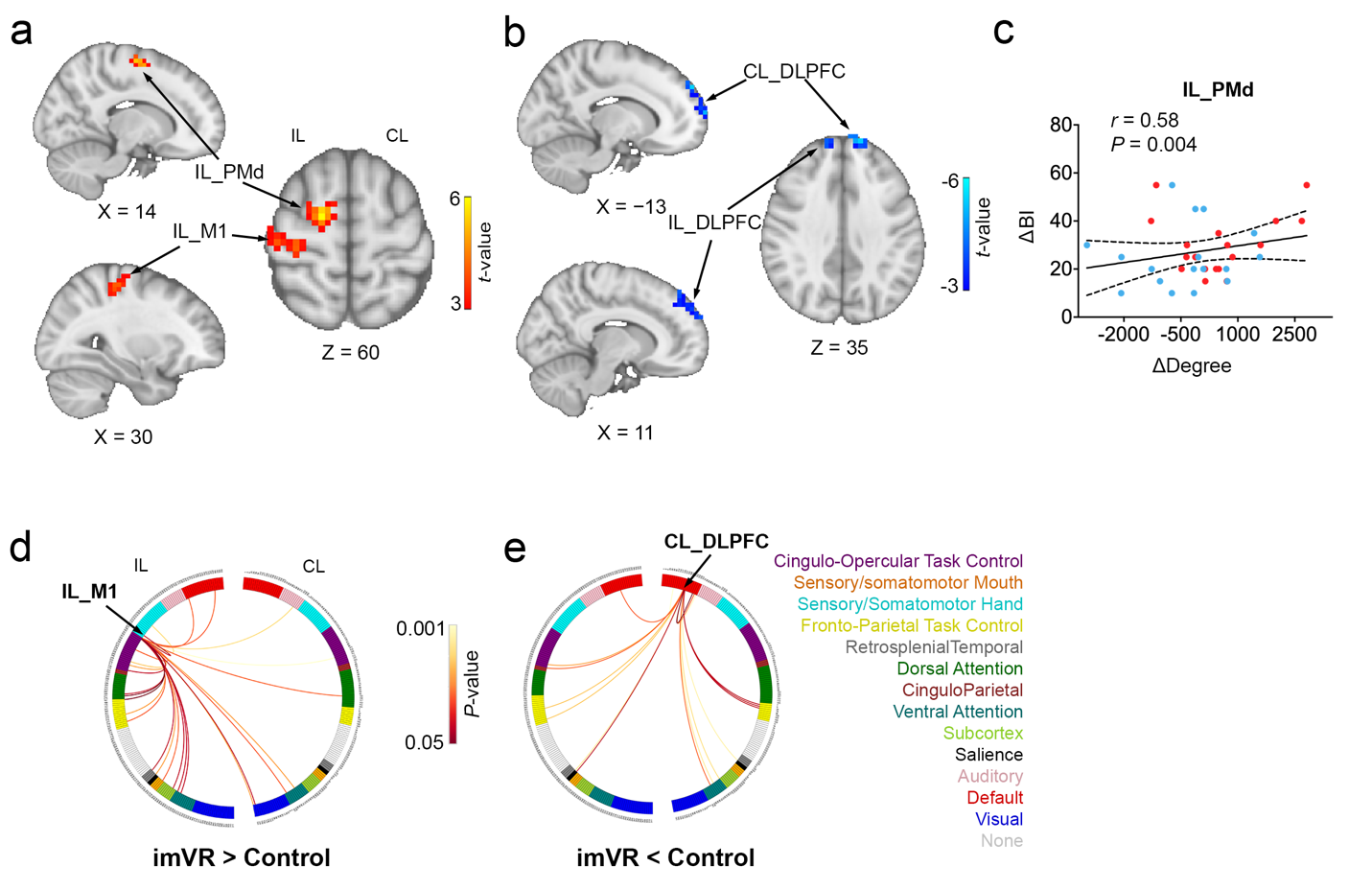
**

**(a)** imVR has greater degree in IL_PMd and IL_M1 compared with the Control group (cluster-corrected, *t* > 3.5, *P*< 0.01). **(b)** imVR has lower degree in CL_ DLPFC and IL_DLPFC regions compared with the Control group (cluster-corrected, *t* > 3.5, *P*< 0.01). **(c)** IL_PMd degree changes positively correlate with changes in BI (*r* = 0.58, *P* = 0.004) from baseline to the end of the intervention. **(d)** The circular plot shows the difference in functional connections to IL_M1 between the imVR and Control groups (*P* < 0.05) in the network space. The IL_M1 region assigned to the sensory/somatomotor hand network. Most of the differences in connections are from the fronto-parietal task control, ventral attention, dorsal attention, default and cingulo-opercular task control networks on the ipsilesional hemisphere. **(e)** The circular plot shows the difference in functional connections to CL_LDPFC between the imVR and Control groups (*P* < 0.05) in the network space. The CL_LDPFC region is assigned to the default mode network (DMN). Most of the differences in connections are from ventral attention, fronto-parietal task control and DMN on the contralesional hemisphere, and DMN, cingulo-opercular task control and frontal-parietal task control network on ipsilesional hemisphere.

ΔDegree = post-intervention degree minus baseline degree; ΔBI = post-intervention BI minus baseline BI; PMd, Dorsal Premotor Cortex; M1, Primary Motor Cortex; DLPFC, Dorsolateral Prefrontal Cortex; IL, Ipsilesional; CL, Contralesional.

**Figure 3 Brain Properties at the End of Follow-up**

**
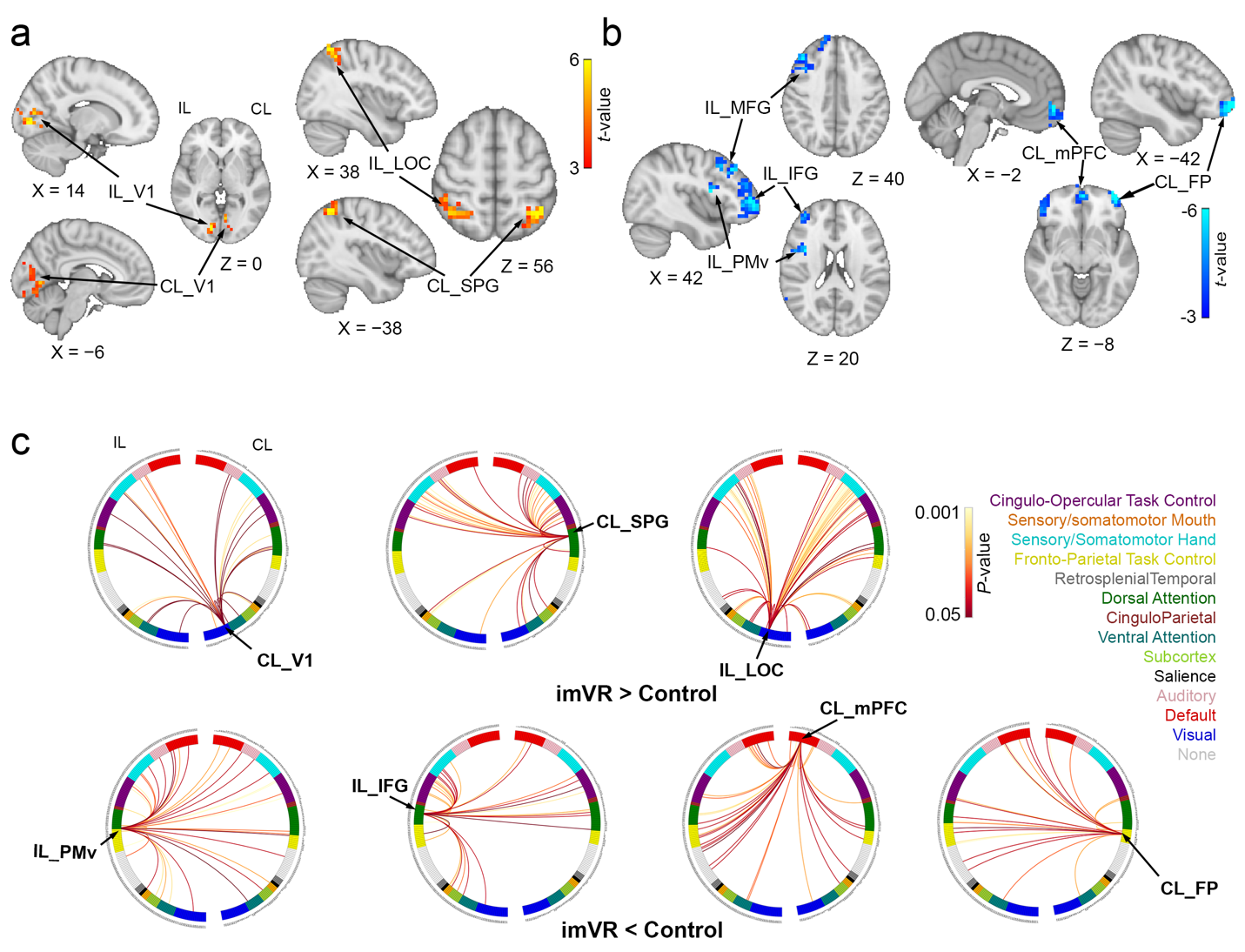
**

**(a)** imVR group has greater degree in IL_V1, CL_V1, CL_SPG and IL_LOC regions at the end of the follow-up compared with the Control group (cluster-corrected, *t* > 3.5, *P*< 0.01). (**b)** imVR group has lower degree in IL_MFG, IL_PMv, IL_ IFG, CL_mPFC and CL_FP regions at the end of intervention compared with the Control group (cluster-corrected, *t* > 3.5, *P*< 0.01). (**c)** Functional connections to CL_V1, CL_SPG and IL_LOC differ between the Control and imVR groups (*P* < 0.05) in the network space. CL_V1 and IL_LOC regions were assigned to the visual network and CL_SPG assigned to the dorsal attention network. Most of the differences of connections are from the sensory/somatomotor hand, visual, auditory, cingulo-opercular task control, dorsal attention and ventral attention networks on the contralesional hemisphere. Circular plot shows the difference in functional connections to IL_PMv, IL_IFG, CL_mPFC and CL_FP, which differed between the Control and imVR groups (*P* < 0.05) in the network space. IL_PMv and IL_IFG assigned to the dorsal attention network and most of the differences in connections are from the default, dorsal attention, cingular-opercular task and control sensory/somatomotor hand networks on the contralesional hemisphere. CL_mPFC and CL_FP were assigned to the default and fronto-parietal task control network, respectively. Most of the differences in connections in both CL_mPFC and CL_FP are from the dorsal attention, default mode network (DMN), fronto-parietal task control, cingular-opercular task control networks on the ipsilesional hemisphere.

V1, Primary Visual Cortex; SPG, Superior Parietal Gyrus; LOC, Lateral Occipital Cortex; MFG, Middle Frontal Gyrus; PMv, ventral premotor cortex; IFG, Inferior frontal gyrus; mPFC, medial Prefrontal Cortex; FP, Frontal Pole; IL, Ipsilesional; CL, Contralesional.

**Figure 4 Six imVR Programs
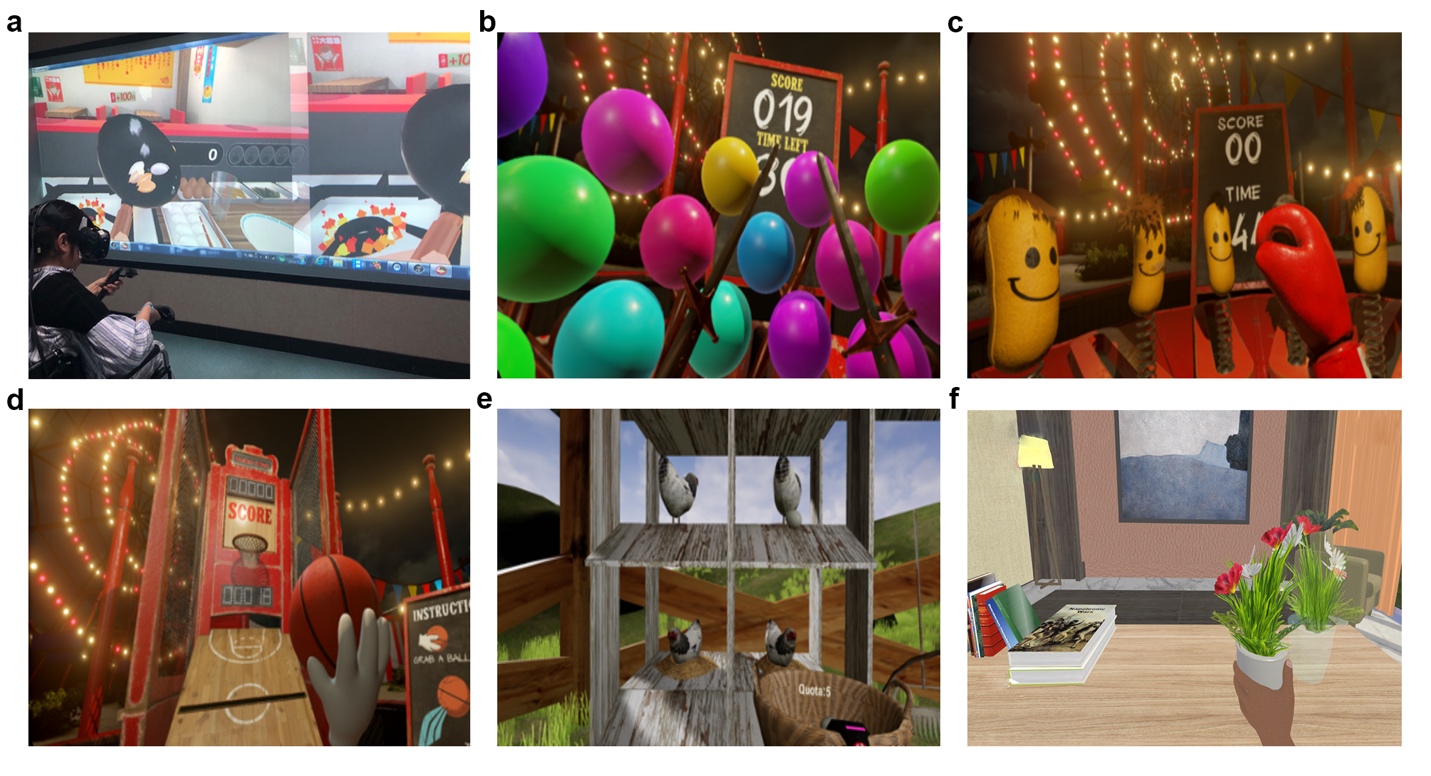
**

**(a)** Frying dumplings and noodles by controlling a wok handle in a virtual kitchen. (**b)** Popping balloons by controlling a sword in a virtual fencing hall. (**c)** Punching with dolls by controlling a big fist in a virtual boxing arena. (**d)** Playing basketball in a virtual court, in which a controller shoots the ball, and the height and distance is varied over time. **(e)** Collecting eggs into a virtual basket by a controller. (**f)** Tidying up a desk and moving objects to a designated position in a virtual office.

**Figure 5** **Comparison of Brain Motion Between the imVR and the Control Groups**


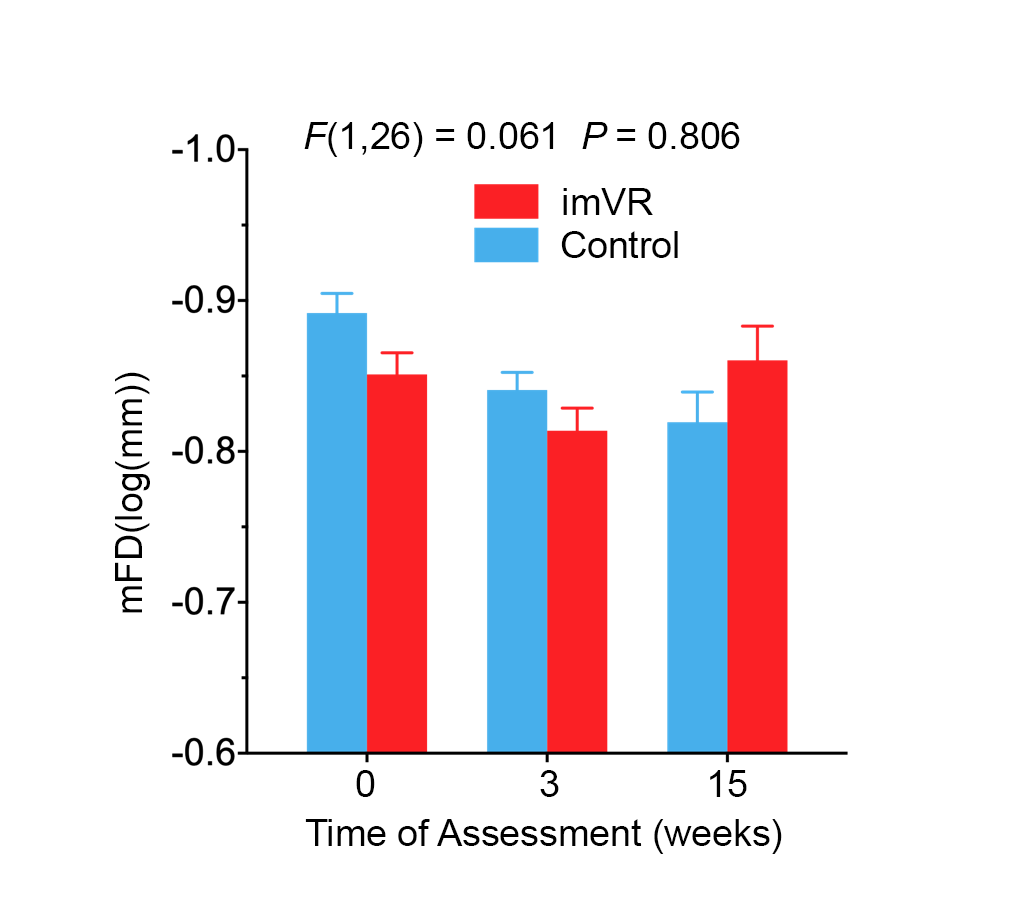


Bar graph (mean and SE) shows no significant difference of brain motion during scanning, represented by *log*(mFD), across three assessments between the imVR and the Control groups. mFD, mean frame-wise displacement.

References

1. Yang, L., et al., *Dissimilarity of functional connectivity uncovers the influence of participant's motion in functional magnetic resonance imaging studies.* Hum Brain Mapp, 2021. **42**(3): p. 713-723.
